# Complete genome sequence of the heavy metal resistant bacterium *Agromyces aureus* AR33^T^ and comparison with related *Actinobacteria*

**DOI:** 10.1186/s40793-016-0217-z

**Published:** 2017-01-05

**Authors:** Erika Corretto, Livio Antonielli, Angela Sessitsch, Stéphane Compant, Christoph Höfer, Markus Puschenreiter, Günter Brader

**Affiliations:** 1 AIT Austrian Institute of Technology, Health and Environment Department, Konrad-Lorenz-Straße 24, A-3430 Tulln, Austria; 2 Department of Forest and Soil Sciences, University of Natural Resources and Life Sciences (BOKU), Konrad-Lorenz-Straβe 24, A-3430 Tulln, Austria

**Keywords:** *Agromyces aureus*, Genome sequence, Comparative genomics, *Microbacteriaceae*, Heavy metals

## Abstract

*Agromyces aureus* AR33^T^ is a Gram-positive, rod-shaped and motile bacterium belonging to the *Microbacteriaceae* family in the phylum *Actinobacteria* that was isolated from a former zinc/lead mining and processing site in Austria. In this study, the whole genome was sequenced and assembled combining sequences obtained from Illumina MiSeq and Sanger sequencing. The assembly resulted in the complete genome sequence which is 4,373,124 bp long and has a GC content of 70.1%. Furthermore, we performed a comparative genomic analysis with other related organisms: 6 *Agromyces* spp., 4 *Microbacteriaceae* spp. and 2 other members of the class *Actinobacteria*.

**Electronic supplementary material:**

The online version of this article (doi:10.1186/s40793-016-0217-z) contains supplementary material, which is available to authorized users.

## Introduction

*Agromyces aureus* AR33^T^ is a type strain belonging to the *Microbacteriaceae* family, *Actinobacteria* phylum [1]. It is a heavy metal resistant bacteria that was isolated from the rhizosphere of a willow tree (*Salix caprea* L.) grown in a heavy metal contaminated site (Arnoldstein, Austria). Among other bacteria isolated from the same source, AR33^T^ was able to significantly increase the extractability of zinc and cadmium from a contaminated soil [2]. Moreover, the inoculation of AR33^T^ in combination with the fungus *Cadophora finlandica* caused an increase of zinc and cadmium concentration in the shoots of *Salix caprea* L. plants growing in a heavy metal contaminated soil [3]. Based on these interesting features and the fact that the *Agromyces* genus is still a relatively unexplored genus, we decided to sequence the whole genome of *A. aureus* AR33^T^ to gain insights in this genus and the heavy metal resistance and immobilization and mobilization mechanisms. At the time of writing (June 2016), 27 species of the *Agromyces* genus have been recognized and only nine draft genomes are available in the NCBI database. Here, we present the first complete genome sequence of an *Agromyces* species, *A. aureus* AR33^T^ and a comparative analysis with other *Agromyces* spp. and related members of the class *Actinobacteria*.

## Organism information

### Classification and features

**Fig. 1:**
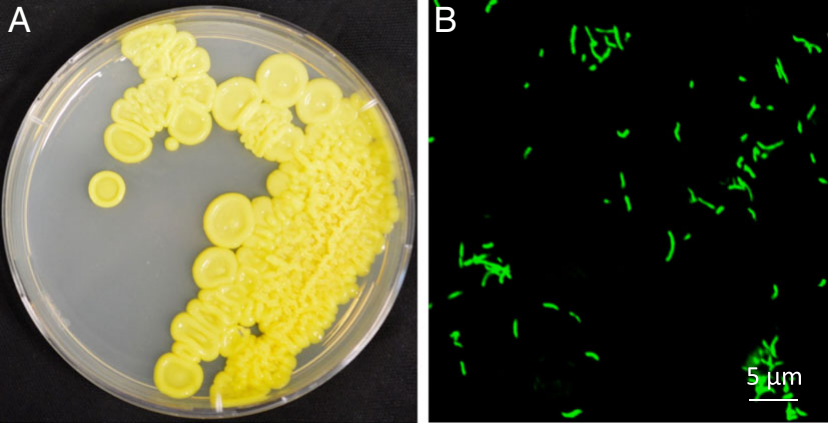
**a**: Picture of *A. aureus* AR33^T^ grown in solid Landy medium; **b**: Confocal laser scanning microscope microphotograph of *A. aureus* AR33^T^. Cells were stained with 3 μM green fluorescent nucleic acid stain SYTO9 (ThermoFisher)

**Fig. 2:**
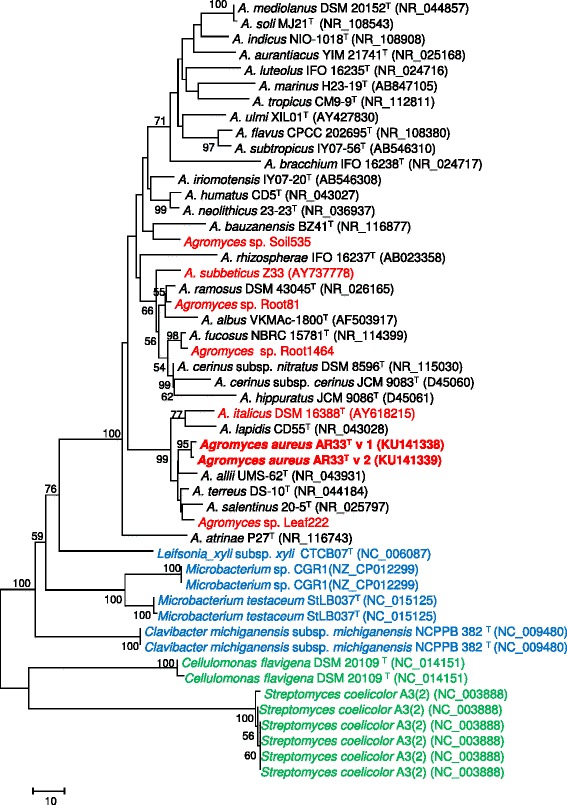
NJ phylogenetic tree based on 16S rRNA gene sequences. GenBank accession numbers are shown in parenthesis. The sequences were aligned with MUSCLE and the phylogenetic tree was calculated in MEGA6 [34] with bootstrap value of 1000 replicates. In bold red *A. aureus* AR33^T^; in red *Agromyces* spp. with published genomes used in this study for further comparison; in blue and green related members of the *Microbacteriaceae* family and of the *Actinobacteria* class used in this study for further comparison, respectively

**Fig. 3:**
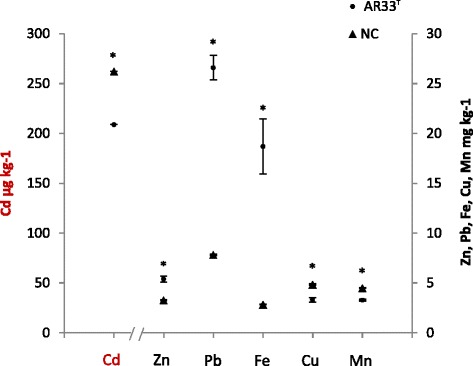
Heavy metal mobilization assays. Contaminated soil was shaken with filtrates of stationary cultures of *A. aureus* AR33^T^ grown in Landy medium (AR33^T^, *n* = 6) and with not inoculated Landy medium (NC, *n* = 3). Significant differences of culture filtrates to control (*p* < 0.05 identified with t-tests) are labeled with an asterisk (*). Error bars show the standard error

*A. aureus* AR33^T^ is a Gram-positive bacterium having yellow-pigmented colonies (Fig. 1a). Cells are rod shaped and can form curved hyphae (Fig. 1b). Phylogenetic analysis based on 16S rRNA genes of other *Agromyces* strains and related members of the same family (*Microbacteriaceae*) and class (*Actinobacteria*) is shown in Fig. 2. The general features of the strain are summarized in Table 1. In order to investigate the potential of *A. aureus* AR33^T^ as plant-associated microbe from a heavy metal contaminated environment, we performed the following additional assays: production of auxins and siderophores, phosphate solubilization, resistance to heavy metals and heavy metal mobilization. To maximize metabolite production necessary for these properties, assays were performed in Landy medium (20 g l^−1^ glucose, 5 g l^−1^ glutamate, 0.25 g l^−1^ MgSO_4_, 0.25 g l^−1^ KCl, 0.5 g l^−1^ KH_2_PO_4_, 150 μg l^−1^ FeSO_4_, 5 mg l^−1^ MnSO_4_, 160 μg l^−1^ CuSO_4_, 1 g l^−1^ yeast extract, pH 7.2) [4], often used for secondary metabolite analysis in gram positive bacteria [5]. The optimal growth temperature and pH values are 28 °C and 6.5–7.5, respectively. AR33^T^ showed oxidase, catalase activity and produced auxins [4, 6]. No phosphate solubilization activity [7] was detected. AR33^T^ is resistant up to 6 mM of zinc and lead and up to 1 mM of cadmium. The production of siderophores was observed using the chrome azurol S assay [8] with Landy (without iron) as growth medium, but not in MM9. The latter is in accordance with a previous study using MM9 [2]. The ability to change the solubility of metals in soil was tested in heavy metal mobilization assays performed as described in [2], but using Landy as growth medium. In Landy, AR33^T^ increased manifold the extractability of lead and iron, whereas the extractability of zinc was slightly increased and the extractability of cadmium, copper and manganese slightly decreased (Fig. 3). Earlier results showed an increase in extractability of both Zn and Cd eased with AR33^T^ in tryptic soy broth [2], suggesting that the production of secondary metabolites such as siderophores and other chelating compounds can be influenced by the growth medium, previously documented for a number of members of the class *Actinobacteria* [9].

**Table 1:** Classification and general features of *Agromyces aureus* AR33^T^

MIGS ID	Property	Term	Evidence code^a^
	Classification	Domain *Bacteria*	TAS [37]
		Phylum *Actinobacteria*	TAS [38]
		Class *Actinobacteria*	TAS [39]
		Order *Micrococcales*	TAS [40]
		Family *Microbacteriaceae*	TAS [40]
		Genus *Agromyces*	TAS [41]
		Species *Agromyces aureus*	TAS [1]
		Type strain: AR33^T^ (=DSM 101731^T^ = LMG 29235^T^)	
	Gram stain	Positive	TAS [1]
	Cell shape	Rod	TAS [1]
	Motility	Motile	TAS [1]
	Sporulation	Not reported	
	Temperature range	10–30 °C	TAS [1]
	Optimum temperature	28 °C	TAS [1]
	pH range; Optimum	5–9; 6,5–7,5	TAS [1]
	Carbon source	Amygdaline, D-glucose, sucrose, L-arabinose and L-rhamnose	TAS [1]
MIGS-6	Habitat	Rhizosphere of *Salix caprea*	TAS [1]
MIGS-6.3	Salinity	Up to 3% NaCl (w/v)	TAS [1]
MIGS-22	Oxygen requirement	Aerobic/microaerophilic	TAS [1]
MIGS-15	Biotic relationship	Free-living	NAS
MIGS-14	Pathogenicity	Unknown	NAS
MIGS-4	Geographic location	Austria: Arnoldstein	TAS [1]
MIGS-5	Sample collection	2001	TAS [1]
MIGS-4.1	Latitude	46.55 N	TAS [1]
MIGS-4.2	Longitude	13.69 E	TAS [1]
MIGS-4.4	Altitude	578 m	TAS [1]

^a^ Evidence codes - IDA: Inferred from Direct Assay; TAS: Traceable Author Statement (i.e., a direct report exists in the literature); NAS: Non-traceable Author Statement (i.e., not directly observed for the living, isolated sample, but based on a generally accepted property for the species, or anecdotal evidence). These evidence codes are from the Gene Ontology project [42]

#### Chemotaxonomic data

*A. aureus* AR33^T^ has a peptidoglycan type B2γ (D-Glu-L-Dab). Galactose, rhamnose, ribose and fucose constitute the cell-wall sugars. The major cellular fatty acids are anteiso-C15:0, anteiso-C17:0 and iso-C16:0, while diphosphatidylglycerol, glycolipid and phosphatidylglycerol are the predominant polar lipids. The main menaquinones are MK-11, −10 and −12.

## Genome sequencing information

### Genome project history

The genome of *A. aureus* AR33^T^ was sequenced by GATC Biotech AG, Konstanz, Germany and subsequently assembled at our institute. The complete genome sequence is available in the NCBI database under the following accession number CP013979. The genome sequencing project information is summarized in Table 2.

**Table 2:** Project information

MIGS ID	Property	Term
MIGS 31	Finishing quality	Complete
MIGS-28	Libraries used	Illumina paired-end library
MIGS 29	Sequencing platforms	Illumina, MiSeq
MIGS 31.2	Fold coverage	259.63X ± 45.98
MIGS 30	Assemblers	SPAdes 3.1.0
MIGS 32	Gene calling method	GeneMarkS+ (PGAAP); Prodigal 2.60 (Prokka)
	Locus Tag	ATC03
	Genbank ID	CP013979
	GenBank Date of Release	09-JUNE-2016
	GOLD ID	–
	BIOPROJECT	PRJNA302856
MIGS 13	Source Material Identifier	AR33^T^
	Project relevance	Genome comparison

### Growth conditions and genomic DNA preparation

*A. aureus* AR33^T^ cells were grown in Landy medium for 48 h at 28 °C with continuous shaking at 200 rpm. DNA was isolated using a phenol-chloroform based protocol. Briefly, cells were collected by centrifugation, re-suspended in lysis buffer (0.1 M NaCl, 0.05 M EDTA pH8, lysozyme 100 mg mL^−1^) and incubated for 10 min at 37 °C. Subsequently, 5% sarkosyl (sodium lauroyl sarcosinate) was added to the solution that was further incubated on ice for 5 min. DNA was extracted using 1 volume of phenol-chloroform-isoamylalcohol (25:24:1) and treated with RNaseA (20 mg mL^−1^) to remove RNA. After an additional cleaning step with chloroform, the DNA was precipitated using 2.5 volumes of ice-cold absolute ethanol and 0.1 volumes of 3 M sodium acetate (pH 5.2) and incubated for 3 h at −20 °C. Genomic DNA was collected by centrifugation; the pellet was washed with 70% ethanol and re-suspended in water. The quality and quantity of DNA were assessed on 1% agarose gel and measured with the NanoDrop spectrophotometer.

### Genome sequencing and assembly

The whole genome was sequenced using the Illumina MiSeq platform (300 bp paired-end reads). Raw reads were screened for PhiX contamination using Bowtie2 [10]. Adapter- and quality-trimming was performed in Trimmomatic-0.32 [11]. Overlapping reads were subsequently merged using FLASH [12] and long single reads and paired end reads assembled with SPAdes 3.1.0 [13]. The initial assembly consisted in 4 contigs, of which one represented the rRNA genes. The gaps between the contigs were closed by designing primers at each contig edge (Additional file 1: Table S1). The PCR products were cloned and sequenced (Sanger). The 4 contigs and the Sanger sequences were manually assembled resulting in a single contig that could be circularized with Circlator [14]. The assembly quality was estimated in QUAST 2.3 [15] and quality control of mapping data performed in Qualimap 1.0 [16]. Phylosift v1.0.1 [17] was used to identify 38 highly conserved, single-copy marker genes that can be used to assess the completeness of the genome [18, 19]. In *A. aureus* AR33^T^ all marker genes could be identified and the phylogenetic analysis showed no contamination. The presence of tRNA genes for all essential amino acids was verified using ARAGORN [20].

### Genome annotation

The *A. aureus* AR33^T^ genome was annotated using the NCBI Prokaryotic Genome Annotation Pipeline as well as Prokka [21, 22]. BLASTClust [23] was used to detect genes in internal clusters with the following threshold parameters: 70% covered length and 30% sequence identity. The COG functional categories were assigned through the WebMGA server [24]. The predicted CDSs were used to search against the Pfam database [25] to assign them to the corresponding protein families. SignalP [26] and TMHMM [27] were used to identify genes containing signal peptides and transmembrane helices, respectively. The detailed information about these features is summarized in Tables 3 and 4.

**Table 3:** Genome statistics

Attribute	Value	% of Total
Genome size (bp)	4,373,124	100.00
DNA coding (bp)	3,961,563	90.60
DNA G + C (bp)	3,065,123	70.09
DNA scaffolds	1	–
Total genes	4005	100.00
Protein coding genes	3928	98.08
RNA genes	77	1.92
Pseudo genes	31	0.77
Genes in internal clusters	1152	28.76
Genes with function prediction	2979	74.38
Genes assigned to COGs	2771	70.54
Genes with Pfam domains	2476	61.82
Genes with signal peptides	326	8.14
Genes with transmembrane helices	1191	29.74
CRISPR	1	0.02

**Table 4:** Number of genes associated with general COG functional categories

Code	Value	%age	Description
J	151	3.84	Translation, ribosomal structure and biogenesis
A	2	0.05	RNA processing and modification
K	264	6.72	Transcription
L	115	2.93	Replication, recombination and repair
B	1	0.03	Chromatin structure and dynamics
D	29	0.74	Cell cycle control, Cell division, chromosome partitioning
V	74	1.88	Defense mechanisms
T	64	1.63	Signal transduction mechanisms
M	139	3.54	Cell wall/membrane biogenesis
N	2	0.05	Cell motility
U	24	0.61	Intracellular trafficking and secretion
O	76	1.93	Posttranslational modification, protein turnover, chaperones
C	154	3.92	Energy production and conversion
G	332	8.45	Carbohydrate transport and metabolism
E	258	6.67	Amino acid transport and metabolism
F	75	1.91	Nucleotide transport and metabolism
H	108	2.75	Coenzyme transport and metabolism
I	100	2.55	Lipid transport and metabolism
P	147	3.74	Inorganic ion transport and metabolism
Q	44	1.12	Secondary metabolites biosynthesis, transport and catabolism
R	351	8.94	General function prediction only
S	261	6.64	Function unknown
–	527	13.42	Not in COGs

The total is based on the total number of protein coding genes in the genome

## Genome properties

**Fig. 4:**
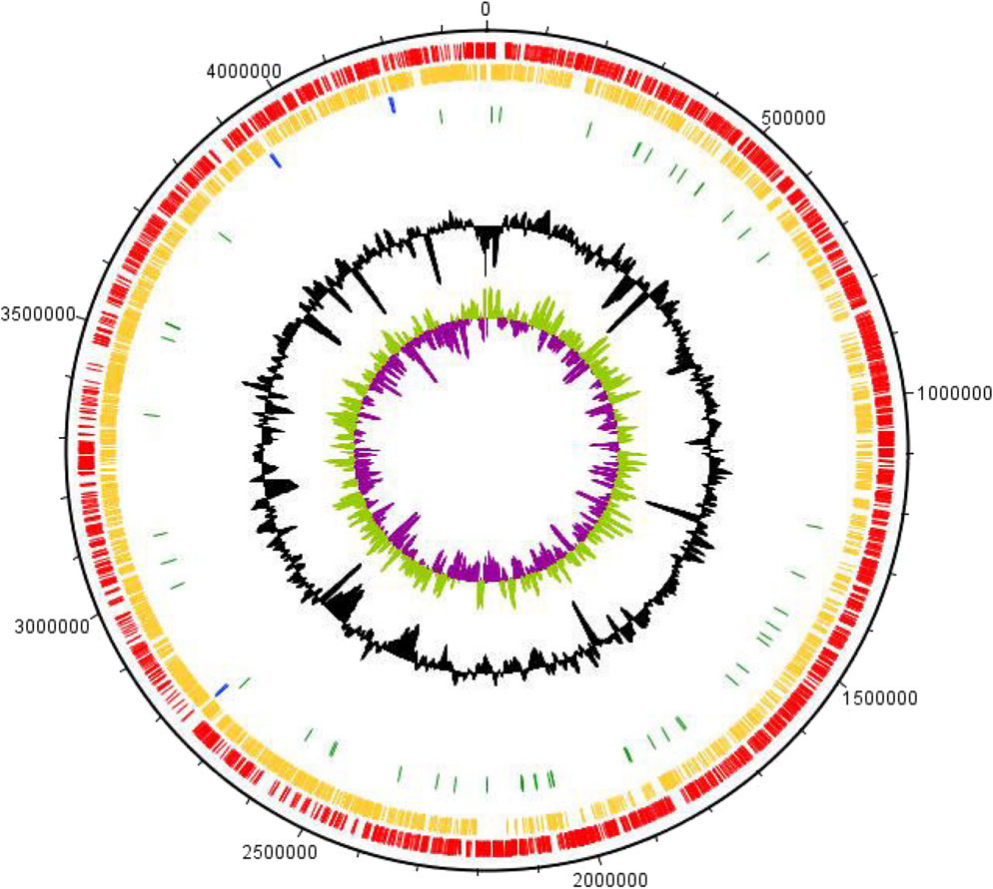
Graphical circular map of *A. aureus* AR33^T^ obtained in DNAPlotter [35]. From inner to outer ring: ring 1 GC skew, ring 2 GC% content, ring 3 tRNAs (*green*), ring 4 rRNAs (*blue*), ring 5 CDSs on reverse strand (*orange*) and ring 6 CDSs on forward strand (*red*)

The complete genome of *A. aureus* AR33^T^ has a total length of 4373124 bp, a CG content of 70.1% and contains three copies of the rRNA operon, of which one has a different 16S rRNA gene sequence (KU141338, KU141339). It has a total of 4005 predicted genes of which 3928 (98.1%) are protein coding genes and 31 are pseudogenes (0.8%). Two thousand nine hundred seventy-nine genes (74.4%) have a functional prediction and 2771 genes (70.5%) could be assigned to a COG functional category (Table 4). Additional information about the genome statistics is shown in Table 3. The map of the genome is represented in Fig. 4.

## Insights from the genome sequence

**Fig. 5:**
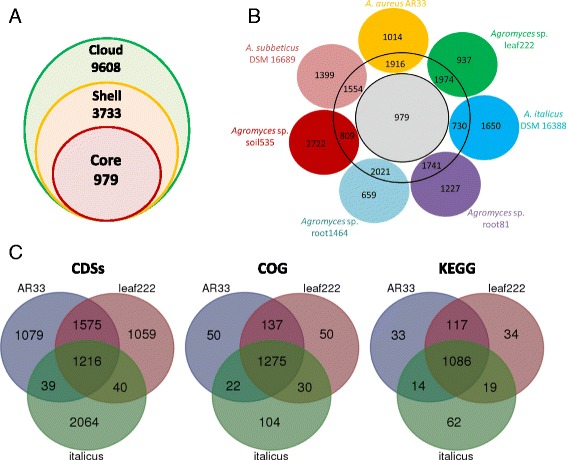
**a**-**b** Pan-genome of *Agromyces* spp. calculated in Roary (blastp 80%) [28]. The inner ring shows the total number of the core genes (present in all the species); the middle ring shows the number of genes in the shell of the pan-genome (present in more than one species); the outer rings show the number of genes in the cloud of the pan-genome (present in only 1 species). **c** Comparison of *A. aureus* AR33^T^ with the closely related species *Agromyces* sp. Leaf222 and *A. italicus* DSM 16388. Venn diagram showing the shared CDSs (Roary, blastp 80%), genes in classified in the same COG functional categories and KEGG metabolic pathways were designed using http://bioinformatics.psb.ugent.be/webtools/Venn/

To gain more information about the genome of *A. aureus* AR33^T^ and about the *Agromyces* genus in general, we performed comparative genomic analysis using other 6 available *Agromyces* genomes with high quality assembly (Table 5). All genomes were annotated in Prokka [22] and the predicted genes were used in Roary [28] to calculate the *Agromyces* pan-genome and core-genome. Since these organisms are members of the same genus but belong to different species, we decided to set the Roary minimum blastp percentage identity at 80%. The choice of this threshold value is supported by the bidirectional best hit analysis performed in RAST [29] (Additional file 1: Figure S1). The *Agromyces* pan-genome has a total of 14,320 genes: 979 represent the core-genome; 3733 and 9608 form the shell and cloud genome, respectively (Fig. 5a). In particular, 1916 genes of *A. aureus* AR33^T^ have orthologues in the shell genome and 1014 genes seem to be unique (Fig. 5b). Subsequently, we focused our comparative analysis on the two closest related organisms with a publicly available genome: *Agromyces* sp. Leaf222 and *A. italicus* DSM 16388 (Fig. 2, Additional file 1: Figure S2). The genome of *A. aureus* AR33^T^ and *Agromyces* sp. Leaf222 seem to be the most similar ones having almost half (1575) of their CDSs sharing at least 80% amino acid similarity. Moreover, these two organisms share 137 COG functional categories and 117 KEGG metabolic pathways (Fig. 5c). Despite being part of the same phylogenetic clade (Fig. 2), *A. italicus* DSM 16388 seems to have a different set of genes and functionalities compared to *A. aureus* AR33^T^ and *Agromyces* sp. Leaf222 (Fig. 5c). Finally, a distinctive feature of the *A. aureus* AR33^T^ genome is the presence of several genes related to metal resistance and homeostasis. For instance, whereas all three have transporters for iron, an essential element, only strain AR33^T^ has transporters also for nickel and cobalt. This feature is probably due to its isolation source, a former zinc/lead mining and processing site, and is in agreement with the displayed ability to mobilize metals (Fig. 3) and to survive in the presence of zinc, lead and cadmium.

**Table 5:** General features of the genomes of *Agromyces* spp. and related organisms used for comparative studies

Organims	Size (Mp)	Plasmids	Contigs	GC%	CDS	rRNA	Isolation source / Characteristics
*A. aureus* AR33	4.37	–	1	70.4	3928	9	*Salix caprea* rhizosphere
*A. italicus* DMS 16388	3.73	–	12	70.2	3370	3	Wall of a tomb
*A. subbeticus* DMS 16689	4.30	–	34	69.1	3947	4	Wall of a cave
*Agromyces* sp. leaf222	4.43	–	4	70.6	3905	5	*Arabidopsis thaliana* leaf
*Agromyces* sp. root81	4.16	–	7	69.7	3959	4	*Arabidopsis thaliana* root
*Agromyces* sp. root1464	4.04	–	3	70.1	3671	5	*Arabidopsis thaliana* root
*Agromyces* sp. soil535	4.83	–	29	70.0	4531	5	Soil
*Microbacterium testaceum* StLB037	3.98	–	1	70.3	3670	6	Potato leaves
*Microbacterium* sp. CGR1	3.63	–	1	68.0	3465	6	Atacama desert, Alto Andino (elevation 4480 m)
*Clavibacter michiganensis subsp. michiganensis* NCPPB 382	3.40	2	1	72.5	3052	6	Phytopathogen of tomato
*Leifsonia xyli subsp. xyli* CTCB07	2.58	–	1	67.7	2722	3	Phytopathogen of sugarcane
*Cellulomonas flavigena* DSM 20109	4.12	–	1	74.3	3742	6	Soil, cellolose- and xylan-degrading
*Streptomyces coelicolor* A3(2)	9.05	2	1	72.0	8316	18	Model representative of soil-dwelling organisms

### Extended insights

**Fig. 6:**
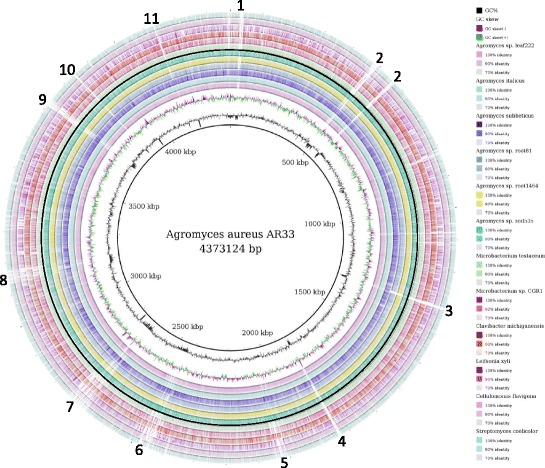
Circular visualization of the whole genome comparison of *A. aureus* AR33^T^, other *Agromyces* spp., related members of the same family and class. The figure was designed using BRIG [31]. The gaps in the circles represent regions of low or no similarity and contain the following features: (1) siderophore biosynthetic gene cluster (desferrioxamine-like); (2) metal related genes like transporters for Pb/Cd/Zn/Hg and for the resistance to As; (3) non-ribosomal peptide synthase modules (pyoverdine-like siderophore) and ABC siderophore transporters; (4) several hypothetical proteins, Mg/Co/Ni transporters, Co/Zn/Cd resistance genes; (5) several hypothetical protein and phage sequences that were detected also in PHAST [36]; (6) Co/Ni transporters, pathway for aromatic compound degradation, transporters for branched chain amino acids; (7) Pb/Cd/Zn/Hg transporters, resistance genes for Cu/Co/Zn/As/Cd, a phage integrase; (8) genes for the production of exopolysaccharides; (9) several hypothetical proteins; (10) Na + H+ antiporters; (11) ABC transporters for Co and heme/siderophore complexes

To obtain further insights into the *A. aureus* AR33^T^ genome, we included related organisms in our comparative analysis: *Microbacterium testaceum* StLB037, *Microbacterium* sp. CGR1, *Clavibacter michiganensis subsp. michiganensis* NCPPB 382, *Leifsonia xyli subsp. xyli* CTCB07, *Cellulomonas flavigena* DSM 20109 and *Streptomyces coelicolor* A3(2). The selection criteria were the following: (i) they have a closed genome; (ii) they are member of the same family (*Microbacteriaceae*) or class (*Actinobacteria*) that have a similar secondary metabolite gene clusters; (iii) they were isolated from soil or are plant-associated bacteria. We performed an all versus all genome comparison in Gegenees [30] to establish the overall similarity of the considered genomes (Additional file 1: Figure S3). The heat map reflects the phylogenetic tree (Fig. 2) and confirms that the closest sequenced relative of *A. aureus* AR33^T^ is *Agromyces* sp. Leaf222. Differences between the analyzed genomes are highlighted in the circular map designed in BRIG [31] (Fig. 6). Interestingly, the gaps indicating regions with low similarity to compared genomes correspond to drastic changes in the GC content of *A. aureus* AR33^T^ and code for: siderophores transporters and biosynthetic clusters, genes related to metal resistance and homeostasis, phage sequences and several hypothetical proteins. A distinctive characteristic of the *Actinobacteria* class is the ability to produce a wide range of secondary metabolites. Therefore, we identified secondary metabolites gene clusters using antiSMASH 3.0 [32] (Table 6). In the *A. aureus* AR33^T^ genome, we could identify a type III PKS gene and clusters for the production of terpenoids, siderophores and lantipeptides. The presence of a siderophore biosynthetic cluster is supported by the positive result in the in vitro CAS assay [8] and could explain the ability to change the mobility of metals like iron and lead demonstrated in the heavy metal mobilization assay. This cluster seems to be involved in the production of a desferrioxamine-like siderophore and is found in other members of the *Microbacteriaceae* family as well. For instance, the genes belonging to the siderophore cluster in *Agromyces* sp. Leaf222 share 79–98% amino acid similarity with the ones of AR33^T^. The terpenoid cluster seems to be widespread among these organisms and is often associated to a yellow pigmentation of the colonies. The type III PKS gene shows similarities to a naringenin-chalcone synthase and is conserved among other *Agromyces* spp. and *Microbacteriaceae* spp. with the exception of *Leifsonia xyli subsp. xyli* CTCB07, which has a longer sequence. Finally, the lantipeptide gene cluster is a rare feature and its structure resembles the one that has been characterized in *Streptomyces venezuelae* for the production of lanthionine-containing peptides [33].

**Table 6:** Secondary metabolite gene clusters identified with antiSMASH [32] in the genomes *Agromyces* spp. and related organisms. Others: cluster containing a secondary metabolite-related protein that does not fit into any other antiSMASH category; putative: putative cluster identified with the ClusterFinder algorithm which is mainly related to saccharides or fatty acids or without a specific prediction

Organims	Siderophore	Terpene	Lantipeptide	T3pks	Others	Putative
*A. aureus* AR33	1	1	1	1	–	35
*A. italicus* DMS 16388	–	–	–	1	1	22
*A. subbeticus* DMS 16689	–	1	–	1	2	29
*Agromyces* sp. leaf222	1	1	–	1	2	39
*Agromyces* sp. root81	–	1	–	1	1	27
*Agromyces* sp. root1464	–	1	–	1	2	25
*Agromyces* sp. soil535	–	–	–	1	3	46
*Microbacterium testaceum* StLB037	–	–	–	1	3	27
*Microbacterium* sp. CGR1	–	1	–	1	1	29
*Clavibacter michiganensis subsp. michiganensis* NCPPB 382	1	1	2	1	4	24
*Leifsonia xyli subsp. xyli* CTCB07	–	1	–	1	1	11
*Cellulomonas flavigena* DSM 20109	1	1	–	1	2	24
*Streptomyces coelicolor* A3(2)	3	5	3	2	16	72

## Conclusions

Heavy metals are recognized as one of the main soil contaminants world-wide. Bacteria such as *A. aureus* AR33^T^ could be used to improve eco-friendly decontamination techniques such as bio-augmentation or phytoremediation. Here, we presented the first complete genome of an *Agromyces* that was isolated from a heavy metal mining/processing site in Austria. It is able to survive in the presence of metals such as zinc, lead and cadmium and can influence the metals mobility of a contaminated soil. Genomic analysis revealed the presence of secondary metabolite gene clusters potentially involved in terpenoid and lantipeptide production, type III PKS and siderophore biosynthesis. In particular, the last two gene clusters could be directly involved in the heavy metal im-mobilization process. Moreover, the correlation between the genotype and phenotype of *A. aureus* AR33^T^ is supported by the presence of several metal resistance and homeostasis genes. We could identify genomic regions displaying low similarity to compared genomes of related organisms, which are characterized by a different GC content and by the presence of genes coding for siderophore transporters and biosynthetic clusters, genes related to metal resistance and homeostasis, phage sequences and several hypothetical proteins. The genome based phylogenetic analysis including closely related and more distant organisms isolated from similar environments appeared to be in agreement with the 16S rRNA gene phylogeny. This brief comparative analysis could be the starting point for further studies in different directions. For instance, it could lead to a deeper understanding of the *Agromyces* genus and its relationship with other members of the class *Actinobacteria* and to a better knowledge about the im-mobilization mechanisms.

## Additional file

Additional file 1:
**Table S1**. Primers used for gap closing. **Figure S1**. Bidirectional best hit analysis performed in RAST. **Figure S2**. Blast Dot Plot of Agromyces aureus AR33 versus Agromyces sp. Leaf222 calculated in RAST. **Figure S3**. Heat map showing similarities between whole genomes of A. aureus AR33 , other Agromyces spp. and related members of the same family and phylum. (PDF 277 kb)

## Abbreviations

ABC: ATP binding cassette
CAS: Chrome azurol S
COG: Clusters of orthologous group
CRISPR: Clustered regularly interspaced short palindromic repeats
Dab: 2,4-diaminobutyric acid
MM9: Minimal medium 9
PKS: Polyketide synthase
