# Supplementary material for: Complete genome sequence of the heavy metal resistant bacterium Agromyces aureus AR33T and comparison with related Actinobacteria
Source: Stand Genomic Sci. 2017 Jan 5;12:2. doi: 10.1186/s40793-016-0217-z (PMC5217419; doi:10.1186/s40793-016-0217-z)
Supplement: Additional file 1: — Table S1. Primers used for gap closing. Figure S1. Bidirectional best hit analysis performed in RAST. Figure S2. Blast Dot Plot of Agromyces aureus AR33 versus Agromyces sp. Leaf222 calculated in RAST. Figure S3. Heat map showing similarities between whole genomes of A. aureus AR33 , other Agromyces spp. and related members of the same family and phylum. (PDF 277 kb) [file 40793_2016_217_MOESM1_ESM.pdf]

Supplementary material to:

Complete genome sequence of the heavy metal resistant bacterium *Agromyces aureus* AR33^{T} and comparison with related Actinobacteria

Erika Corretto,[1] Livio Antonielli,[1] Angela Sessitsch,[1] Stéphane Compant[1], Christoph Höfer[2], Markus Puschenreiter[2] and Günter Brader[1]

[1] AIT Austrian Institute of Technology, Health and Environment Department, Konrad-Lorenz-Straße 24, A-3430 Tulln, Austria

[2] University of Natural Resources and Life Sciences (BOKU), Department of Forest and Soil Sciences, Konrad-Lorenz-Straβe 24, A-3430 Tulln, Austria

Corresponding author: guenter.brader@ait.ac.at

Table S1

Table S1. Primers used to close the gaps between the four contigs from the preliminary assembly. First, primers were designed at the edges (start/end) of each contig. In this way, we could determine that contig C4 (containing the rRNA genes) was positioned at the start and end of the other three contigs. Then, the three gaps (circa 6 kb) were cloned and sequenced with the specific primers listed in this table.

Primers	Sequence 5'-3'
C1-start	GGTGTCGTTGCGTATGTTTA
C1-end	TTCTCATACGGACGCTTGTT
C2-start	GAGGAGGTTGGTGTGTGTTA
C2-end	ATCCCCACCATCTCTTGTTC
C3-start	GTGTTGTAGTTCATGCCGTC
C3-end	TGATAACCCCCATTTCCTGA
C4-start	CGGAAACACAAACACACAAC
C4-end	CCTCACCAACAAGCTGATAG
**Gap C1-end/C2-end**	**Sequence 5'-3'**
C1a-end	CTCTGGGATAAGCGTTGGAA
C2a-end	CACTTTCGTGTGTGTGTAGT

C1b-end	ATGACTCCACCAACACTAGC
C2b-end	CCGCAAGGCTAAAACTCAA
C1c-end	CAAGTTGGTTCACGCCTTAG
C2c-end	GGTCTGGTTTAGTACGCCTC
C1d-end	ACCTGTAATGTCAATGCCAC
C2d-end	CATCTCAGTACCCACAGGAA
Gap C2-start/C3-end	Sequence 5’-3’
C2a-start	TCTCACGACGTTCTAAACCC
C3a-end	TCCTTGACGGTACTTGCAG
C2b-start	CCGTATACATCGTCTTGCGA
C3b-end	GACGTCAAATCATCATGCCC
C2c-start	CTTGACCAGTGAGCTATTAC
C3c-end	GGAACATTGAACATAGATGC
C2d-start	CGCTACTTGGGATACCTCTC
C2e-start	GCCCGAGCATCTATGTTCAA
Gap C1-start/C3-start	Sequence 5’-3’
C1a-start	CTTGTTGGTGAGGTAATGG
C3a-start	GTCTTAGCTTCCGGGTTC
C1b-start	CAAGGCTAAAACTCAAAGGA
C3b-start	GATCCTCTCAAATTTCCTGC
C1c-start	TGGGATCGGTGATTAGGAC
C3c-start	CTACACAAGCCACACCGAAC
C1d-start	GTCAATTTCTCCGGCACTTC
C3d-start	TCATGCCTGCATTCTCACTC

Supplementary figure legends

Figure S1. Bidirectional best hit analysis performed in RAST [29].

Figure S2. Blast Dot Plot of *Agromyces aureus* AR33^T versus *Agromyces* sp. Leaf222 calculated in RAST [29].

Figure S3. Heat map showing similarities between whole genomes of *A. aureus* AR33^T, other *Agromyces* spp. and related members of the same family and phylum. The analysis was performed in Gegenees [30]. The color profile goes from green (high similarity) to red (low similarity).

Figure S1

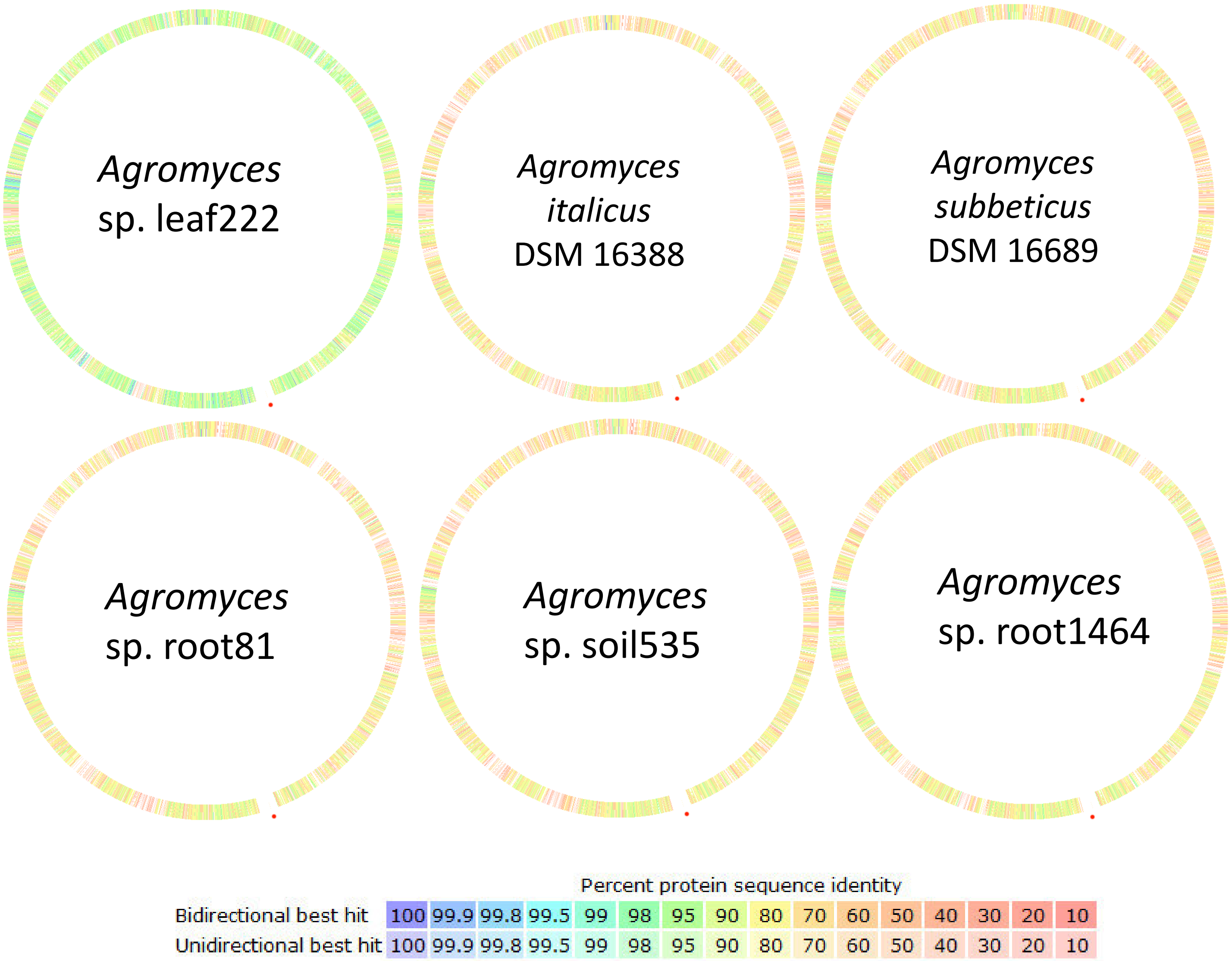

Agromyces sp. leaf222
Agromyces italicus DSM 16388
Agromyces subbeticus DSM 16689
Agromyces sp. root81
Agromyces sp. soil535
Agromyces sp. root1464
Percent protein sequence identity
Bidirectional best hit 100 99.9 99.8 99.5 99 98 95 90 80 70 60 50 40 30 20 10
Unidirectional best hit 100 99.9 99.8 99.5 99 98 95 90 80 70 60 50 40 30 20 10

Figure S2

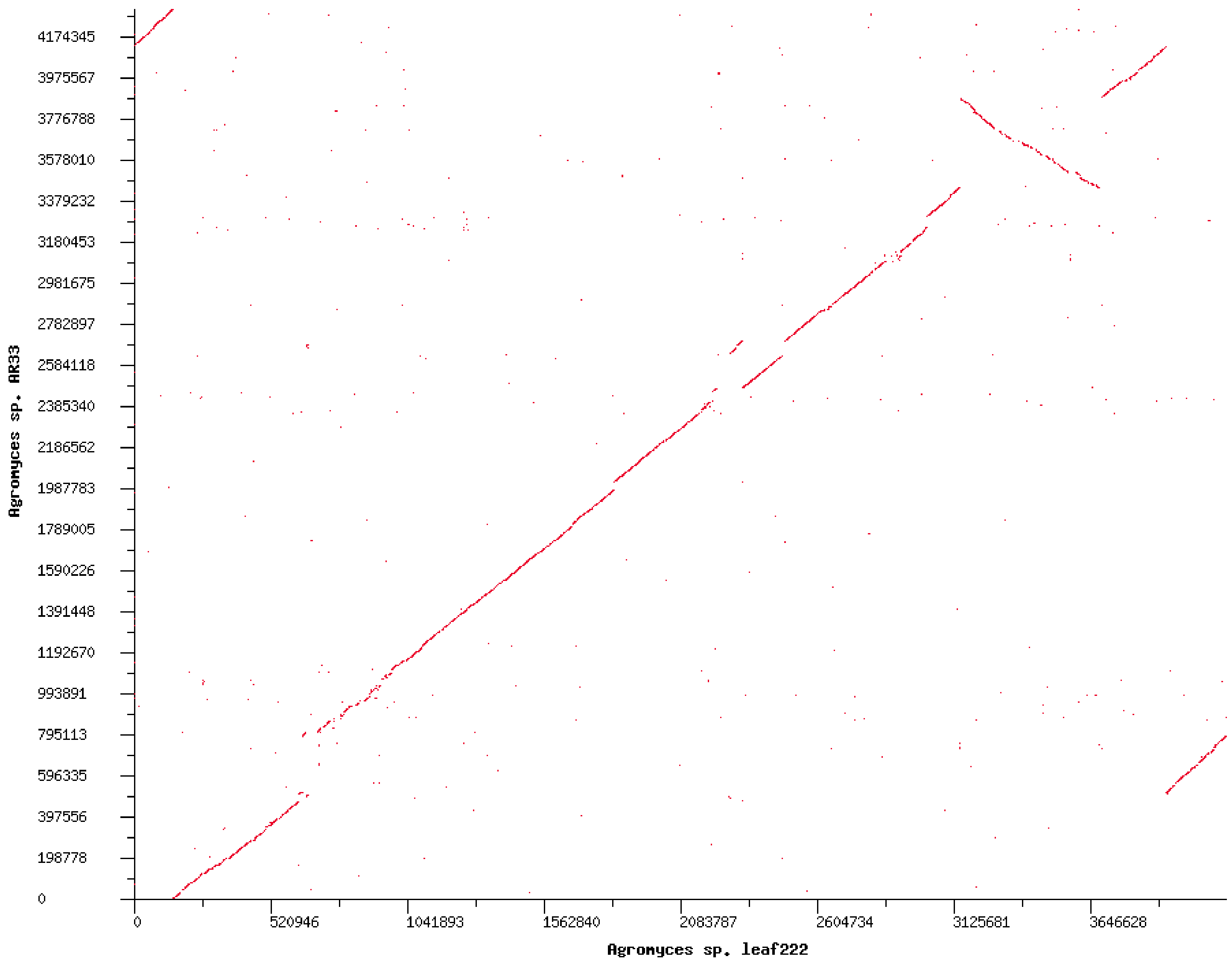

4174345
3975567
3776788
3578010
3379232
3180453
2981675
2782897
2584118
2385340
2186562
1987783
1789005
1590226
1391448
1192670
993891
795113
596335
397556
198778
0
Agromyces sp. AR33
0
520946
1041893
1562840
2083787
2604734
3125681
3646628
Agromyces sp. leaf222

Figure S3

Organisms	1	2	3	4	5	6	7	8	9	10	11	12	13
1: *A. aureus* AR33	100	73	61	61	61	59	60	52	52	52	53	47	43
2: *Agromyces* sp. leaf222	73	100	60	62	62	60	60	51	51	51	53	46	42
3: *A. italicus* DMS 16388	60	60	100	62	62	60	60	52	51	51	52	45	42
4: *Agromyces* sp. root81	61	62	62	100	72	61	66	51	51	51	52	46	42
5: *Agromyces* sp. root1464	61	62	61	72	100	61	67	50	50	51	52	46	42
6: *Agromyces* sp. soil535	59	60	59	61	61	100	60	50	50	51	52	45	41
7: *A. subbeticus* DMS 16689	60	60	60	67	67	60	100	50	50	51	51	45	41
8: *Microbacterium testaceum* StLB037	52	52	52	52	51	51	51	100	55	50	52	46	43
9: *Microbacterium* sp. CGR1	51	52	51	51	51	51	50	55	100	50	52	47	44
10: *Clavibacter michiganensis* subsp. *michiganensis* NCPPB 382	52	52	52	52	52	51	52	50	50	100	53	46	43
11: *Leifsonia xyli* subsp. *xyli* CTCB07	52	53	52	52	52	52	52	50	50	51	100	46	43
12: *Cellulomonas flavigena* DSM 20109	46	47	47	47	47	46	46	47	47	46	47	100	46
13: *Streptomyces coelicolor* A3(2)	44	45	46	46	45	45	45	46	46	45	48	48	100
